# Secretome-Assisted Regenerative Therapy for Hyaluronic Acid Filler-Induced Vascular Occlusion: A Case Report

**DOI:** 10.7759/cureus.101978

**Published:** 2026-01-21

**Authors:** Jassive Sanchez Tovar, Jose L Fregoso Sandoval

**Affiliations:** 1 Aesthetic Medicine, Lilova Aesthetics Institute, Ciudad Guzmán, MEX

**Keywords:** hyaluronic acid fillers, mesenchymal stem cells, regenerative medicine, secretome, vascular occlusion

## Abstract

Hyaluronic acid (HA) fillers are widely used for non-surgical facial contouring, but vascular occlusion remains one of the most feared complications due to its potential to cause necrosis, functional impairment, and permanent deformities. Conventional management focuses on high-dose hyaluronidase and supportive therapy, which may not always prevent long-term sequelae. In recent years, regenerative approaches such as secretome and mesenchymal stem cell (MSC) therapies have emerged as promising alternatives. We report the case of a 29-year-old woman who developed nasal vascular occlusion following HA filler injection by a non-medical injector. Initial presentation included localized pain and cutaneous necrosis in the nasal tip. Early management involved repeated hyaluronidase infiltration, oral sildenafil, a single dose of prednisone, and adjuvant intravenous peroxidation therapy. Subsequently, 12 local applications of standardized MSC-derived secretome were administered. This strategy promoted granulation tissue formation, re-epithelialization, and partial recovery of skin integrity. However, clinical persistence of nasal asymmetry suggested cartilage involvement. To address this, an additional injection of 10 million MSCs was performed, leading to stable cutaneous coverage and improvement of nasal contour within 45 days. At the two-year follow-up, the patient showed a stable scar with mild hypopigmentation and surface irregularity. This case highlights the translational potential of regenerative therapies as an alternative to reconstructive surgery in filler-induced vascular complications. Secretome demonstrated efficacy in soft tissue repair, while MSCs contributed to structural restoration. Regenerative strategies may expand the therapeutic arsenal for HA filler-related vascular events, offering non-surgical and biologically targeted options for tissue preservation and functional recovery.

## Introduction

Hyaluronic acid (HA) dermal fillers have become an increasingly popular minimally invasive alternative in aesthetic medicine due to their ability to remodel facial contours, smooth wrinkles, and provide rapid results with reduced recovery times [1]. In the most recent International Society of Aesthetic Plastic Surgery (ISAPS) Global Survey (procedures performed in 2024), nearly 38 million aesthetic procedures were reported worldwide, including approximately 20.5 million non-surgical procedures, with injectables remaining among the most commonly performed non-surgical treatments [2].

Latin America remains a highly active region for aesthetic medicine. Based on ISAPS country-level volumes, Brazil and Mexico consistently rank among the leading countries worldwide for aesthetic procedures. The regional demand has supported the broad adoption of non-surgical interventions, including HA-based nasal contouring in routine practice [2,3].

Non-surgical rhinoplasty has been described as effective, predictable, and relatively safe, with high patient satisfaction rates [4]. However, despite being considered minimally invasive, the technique is not free of adverse events and complications. The most frequent adverse events include edema, erythema, hematomas, and asymmetries, which are generally mild and self-limiting. The most significant complications, however, include cutaneous necrosis, vision loss, and, in extremely rare cases, cerebral infarction, particularly when nasal or orbital vasculature is compromised [5]. Mechanistically, ischemic injury may result from inadvertent intravascular injection, embolization, or extrinsic compression of end-arterial territories, and the nasal region is considered high-risk due to its vascular anatomy and potential anastomoses with the ophthalmic circulation [5,6].

Cases of localized skin necrosis and progressive ulceration with permanent scarring have been described, and recent reports have even documented structural involvement of the nasal cartilage, which can lead to functional or aesthetic collapse of the nasal pyramid [1,7]. In addition, Wang et al. reported imminent skin necrosis after non-surgical rhinoplasty and suggested that early interventions can help prevent severe complications in the nasal dorsum [1].

The current management of vascular occlusion caused by HA dermal fillers is based on immediate intervention: stopping the procedure, assessing perfusion, performing massage or applying heat as tolerated, and, most importantly, administering high-dose, repeated hyaluronidase, ideally guided by ultrasound when available [7]. This approach can reverse ischemia in up to 80% of cases if initiated within four to six hours [7]. Hyaluronidase remains the cornerstone of treatment to prevent necrosis, with total resolution rates of up to 78% [8]. Supportive measures include antiplatelet agents, vasodilators, corticosteroids, and, in some cases, hyperbaric oxygen therapy [1]. Emerging ultrasound-guided protocols may further improve targeting, confirm reperfusion, and reduce uncertainty in complex presentations [7].

Nevertheless, even with timely intervention, delayed complications may occur due to compression or microocclusion, developing days later and manifesting as progressive skin changes, chronic ulceration, or permanent scarring [7]. Exceptionally, lesions with septal cartilage necrosis following columellar artery occlusion have been described, confirmed by histopathological evaluation in a recent clinical case [9].

The limitations of conventional management have driven the exploration of therapies that, in addition to reversing acute ischemia, also promote tissue regeneration and reduce functional and aesthetic sequelae. In this context, the secretome derived from mesenchymal stem cells (MSC-secretome; MSC-conditioned media) has gained increasing attention, as it represents a complex mixture of bioactive molecules and extracellular vesicles capable of modulating repair and regenerative processes. This secretome has been shown to contain growth factors, cytokines, and extracellular matrix proteins with pro-angiogenic, immunomodulatory, and antifibrotic properties, making it a key component of cell-free therapies [10]. Its potential in skin repair has been documented in systematic reviews showing enhanced angiogenesis, cell migration, and collagen remodeling in difficult-to-heal wounds [11]. In parallel, its translational value in regenerative medicine and dermatology has been highlighted, with reported applications in wound healing and tissue regeneration [12]. More recently, its ability to modulate dermal homeostasis and contribute to skin rejuvenation has been described, providing evidence of its role in preventing skin aging [13]. Although no large-scale clinical reports currently exist regarding its use in filler-induced vascular complications, the available findings suggest that the secretome may represent a promising tool to enhance perfusion and support regeneration in critical areas such as the nasal region.

This article describes the clinical case of a patient who developed vascular compromise following HA dermal filler injection in the nasal region, performed by a non-medical injector dedicated to aesthetic services. The therapeutic approach combined conventional interventions with an innovative regenerative strategy based on the use of secretome. The clinical course and outcomes are detailed, providing preliminary evidence for the potential role of secretome as a non-surgical, biologically targeted therapeutic alternative aimed not only at reversing acute ischemic damage but also at supporting tissue regeneration and preserving both function and aesthetics in filler-associated vascular complications.

## Case presentation

A 29-year-old woman with no relevant medical history and no use of chronic medications or supplements attended an aesthetic service for non-surgical rhinoplasty with an HA dermal filler administered by a non-medical injector. Within the first few hours after the procedure, she developed progressive pain at the injection site, accompanied by localized skin color change at the nasal tip. Neither the patient nor the injector reported livedo reticularis, mottling, or other vascular patterns typically associated with cutaneous ischemia. Despite these early findings suggestive of vascular compromise, no rescue maneuvers or immediate pharmacologic treatment were performed.

**Figure 1:**
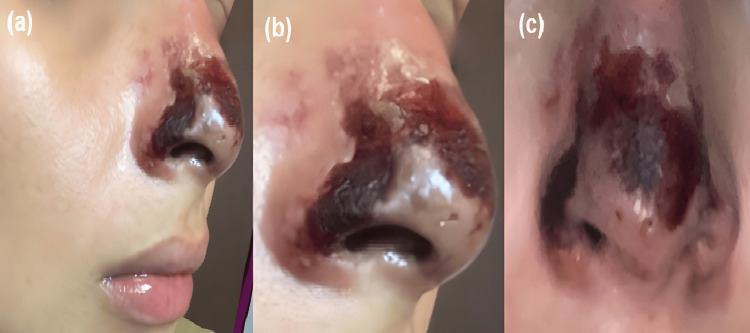
Initial clinical images. Photographs provided by the patient after hyaluronic acid dermal filler injection. (a) Cutaneous necrotic areas are observed in the nasal tip, accompanied by erythema and hemorrhagic crusts on the nasal dorsum. (b) Right lateral view showing central necrosis with progressive border delimitation, associated with local inflammation. (c) Close-up of the nasal tip demonstrating more extensive cutaneous necrosis with central blackish eschar and peripheral erythema, findings consistent with vascular compromise secondary to dermal filler injection.

Several days later, the patient was referred to our clinic for specialized evaluation. At the initial consultation, she had not received any therapeutic intervention, and only photographic documentation of the early evolution was available, provided by the patient to assess the feasibility of medical care (Figure 1). On physical examination, cutaneous lesions with necrotic areas were observed at the nasal tip, along with erythema and hemorrhagic crusts on the nasal dorsum. Localized tenderness was elicited on palpation, supporting ongoing vascular compromise. Taken together, these findings were consistent with vascular occlusion secondary to HA dermal filler injection.

At the time of the first evaluation, a clinical diagnosis of vascular occlusion secondary to HA infiltration was confirmed.

The therapeutic approach was designed according to international recommendations for the management of filler-related vascular complications, which include early and repeated administration of high-dose hyaluronidase, ideally guided by ultrasound when available [7,14,15]. In this case, ultrasound guidance was not employed due to a lack of availability in the treatment center, and the applications were performed clinically. A total of three hyaluronidase infiltrations were administered (1,500 IU each, reconstituted in 0.9% saline solution), every eight hours within the first 24 hours, applied directly to the affected area with the objective of reversing the vascular compromise [16].

As an initial adjuvant, oral sildenafil 50 mg was prescribed to promote microvascular vasodilation and optimize tissue perfusion, given that the use of phosphodiesterase-5 inhibitors is supported by evidence in models of cutaneous ischemia and flap viability [17,18], and there are reports of filler-induced occlusion successfully managed with a drug of the same class in combination with hyaluronidase [19]. Concomitantly, a single low dose of prednisone (5 mg) was administered on the first day to modulate the inflammatory response, a measure described as an optional adjuvant in guidelines and reviews of filler-related vascular events [15,20].

Subsequently, an intravenous peroxidation therapy protocol was initiated for three consecutive days, consisting of hydrogen peroxide 3.5% diluted in 250 mL of saline solution, combined with 3 cm³ of dimethyl sulfoxide (DMSO), administered over approximately 90 minutes per infusion, twice daily. The rationale was to enhance tissue oxygenation and generate a stimulatory microenvironment for wound healing. The biological support is based on the fact that hydrogen peroxide, at controlled concentrations, can act as a transient oxidative signaling molecule that activates pathways involved in cutaneous repair and angiogenesis [21,22]. In addition, DMSO possesses antioxidant and vasodilatory properties and enhances the penetration of molecules into tissues, which makes it useful in regenerative dermatology [23].

Subsequently, standardized secretome infiltrations (2 mL, 250 µg) were administered, initially impregnated in an electrolyzed superoxidized solution, with the aim of promoting tissue regeneration in the affected area. During the first week, three doses were applied, followed by a progressive scheme that completed a total of 12 applications throughout the treatment period. The secretome, defined as the collection of soluble proteins, growth factors, cytokines, and extracellular vesicles released by MSCs, has been recognized as a key mediator in tissue regeneration by modulating processes of angiogenesis, immunoregulation, and dermal remodeling [10,11].

**Figure 2:**
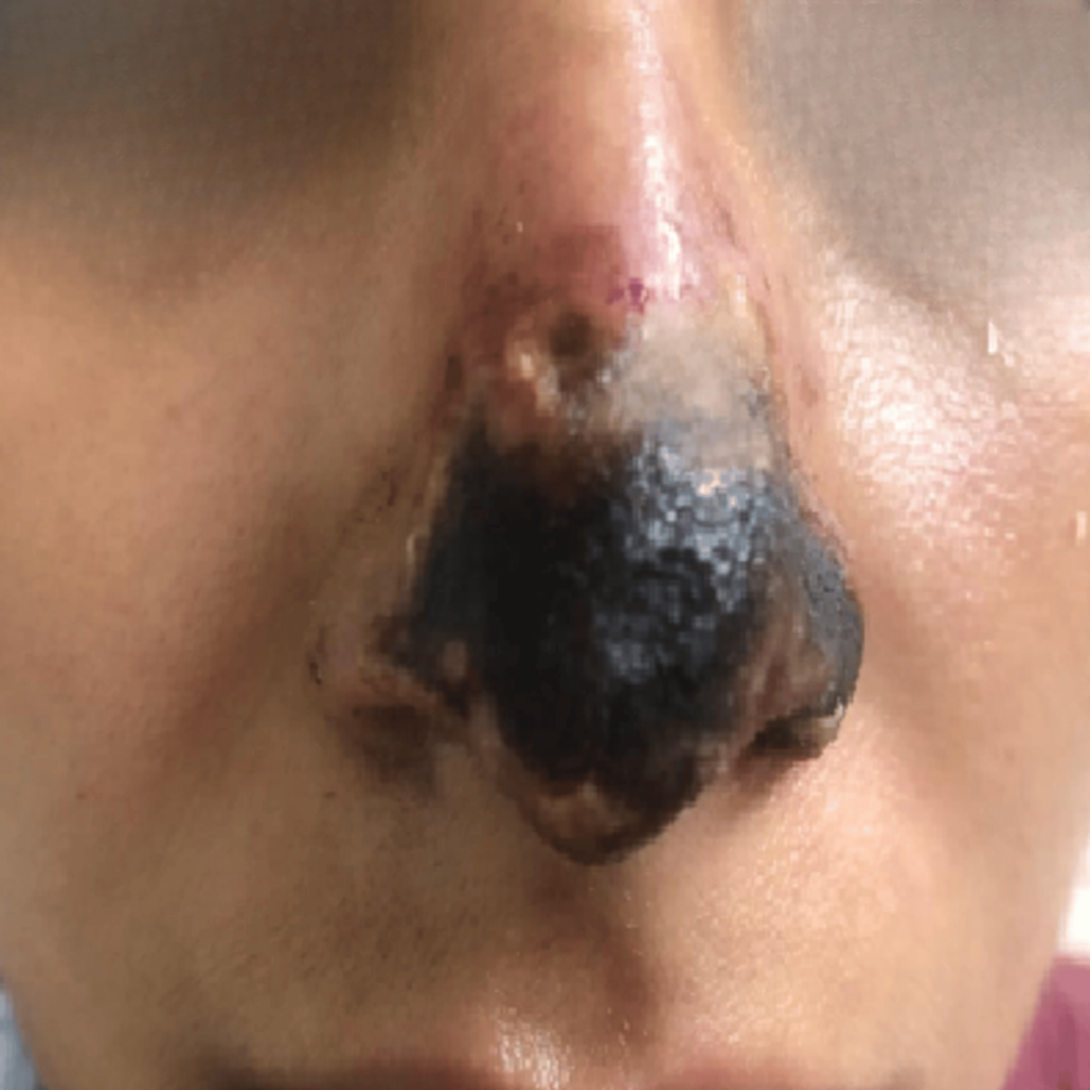
Clinical evolution on day six of follow-up (11 days after the initial procedure). The nasal tip shows a dry, well-demarcated necrotic eschar with partial extension toward the dorsum and erythematous borders. The lesion presents central adherence and absence of signs of active infection, findings consistent with cutaneous necrosis secondary to hyaluronic acid filler-induced vascular occlusion.

On the sixth day of follow-up at our clinic, corresponding to day 11 after the initial filler injection, the patient presented with a well-demarcated necrotic lesion in the nasal tip, extending partially toward the dorsum, with erythematous borders and localized tenderness upon palpation (Figure 2). The necrotic area exhibited a dry, adherent eschar without signs of active infection. The therapeutic strategy was continued, avoiding surgical debridement to preserve the tissue as a biological scaffold for subsequent regeneration.

**Figure 3:**
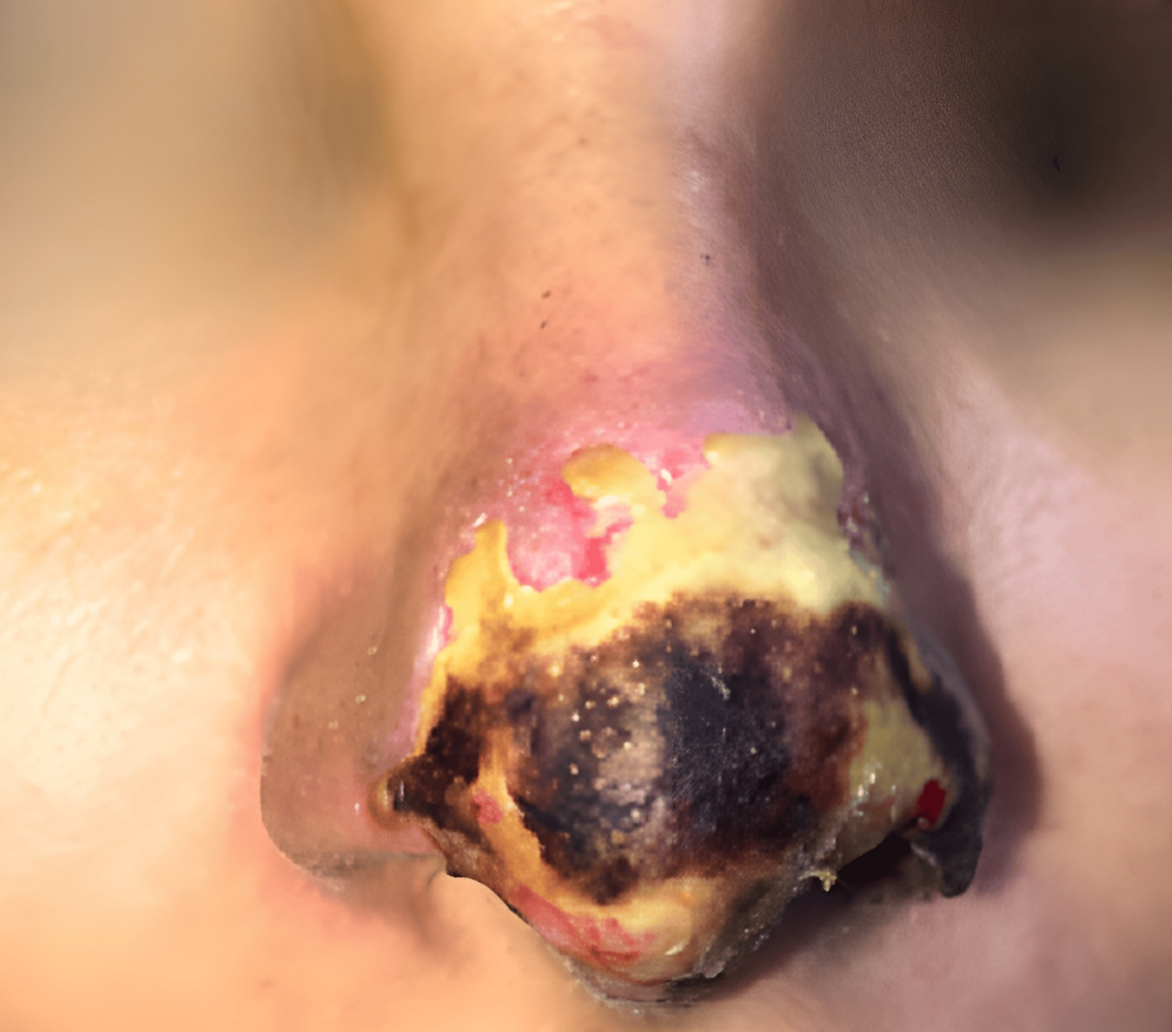
Clinical evolution on day 13 of follow-up (18 days after the initial procedure). The nasal tip shows persistence of a central necrotic eschar with peripheral erythematous tissue and early signs of marginal detachment. Granulation tissue is evident at the periphery, suggesting activation of the reparative process, with no clinical evidence of active infection.

At day 13 of follow-up in our clinic, corresponding to day 18 after the initial filler injection, the patient presented with persistence of a central necrotic eschar on the nasal tip, accompanied by peripheral erythematous areas and early signs of marginal detachment. Incipient granulation tissue was observed at the lesion’s periphery, indicative of activation of the reparative process. No clinical signs of active infection were noted at this stage of the evolution (Figure 3).

**Figure 4:**
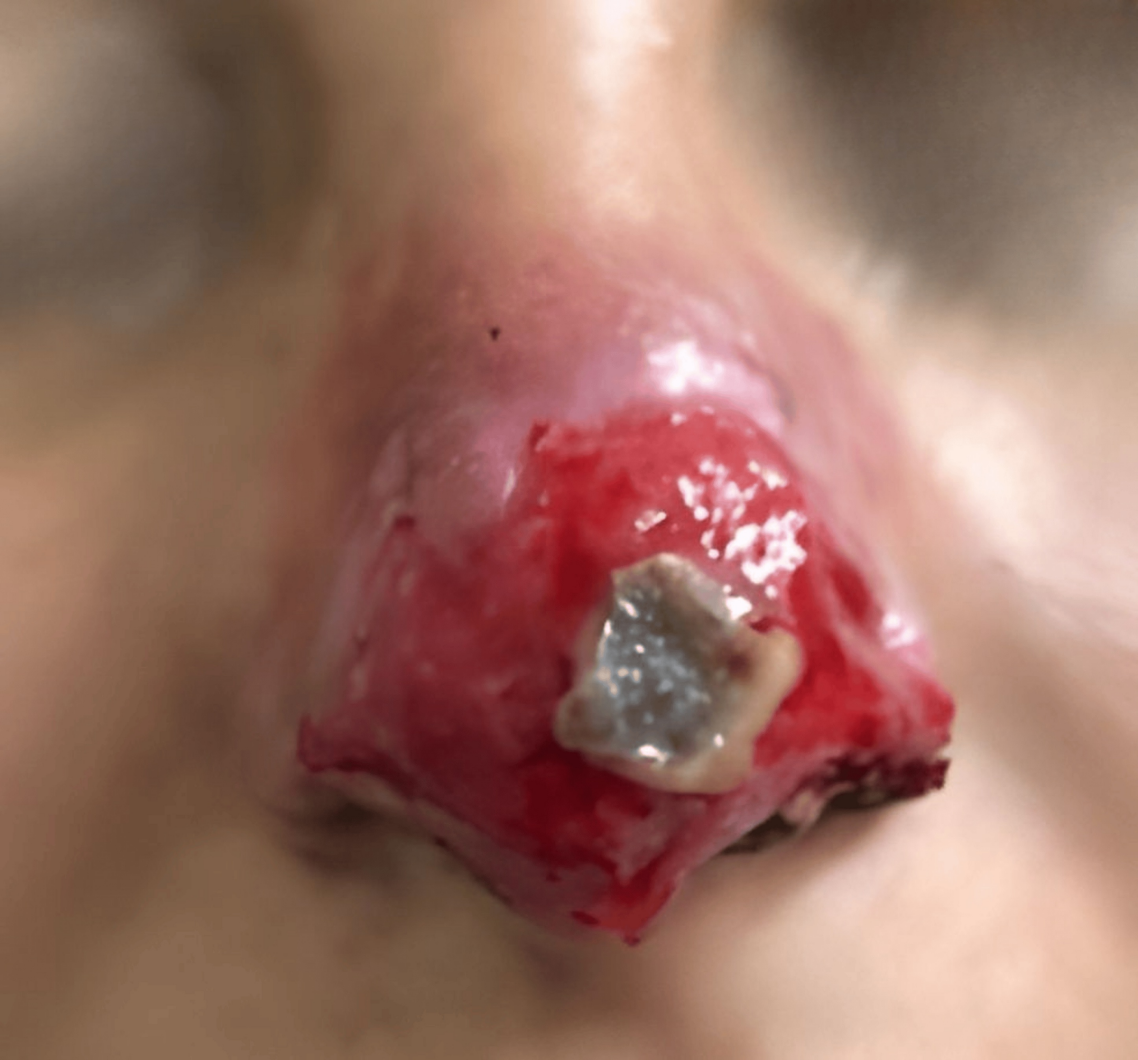
Clinical evolution on day 20 of follow-up (25 days after the initial procedure). Partial detachment of the necrotic eschar with exposure of an erythematous, moist bed rich in granulation tissue. Central remnants of devitalized tissue remain in spontaneous resolution, with no evidence of active infection.

At the evaluation on day 20 of follow-up (25 days after the initial procedure), partial detachment of the necrotic eschar was observed, exposing an erythematous and moist wound bed characterized by abundant granulation tissue. Small fragments of devitalized tissue persisted in the central area, undergoing spontaneous elimination. No clinical signs of infection were present, and the patient reported progressive improvement in pain and local sensitivity (Figure 4).

**Figure 5:**
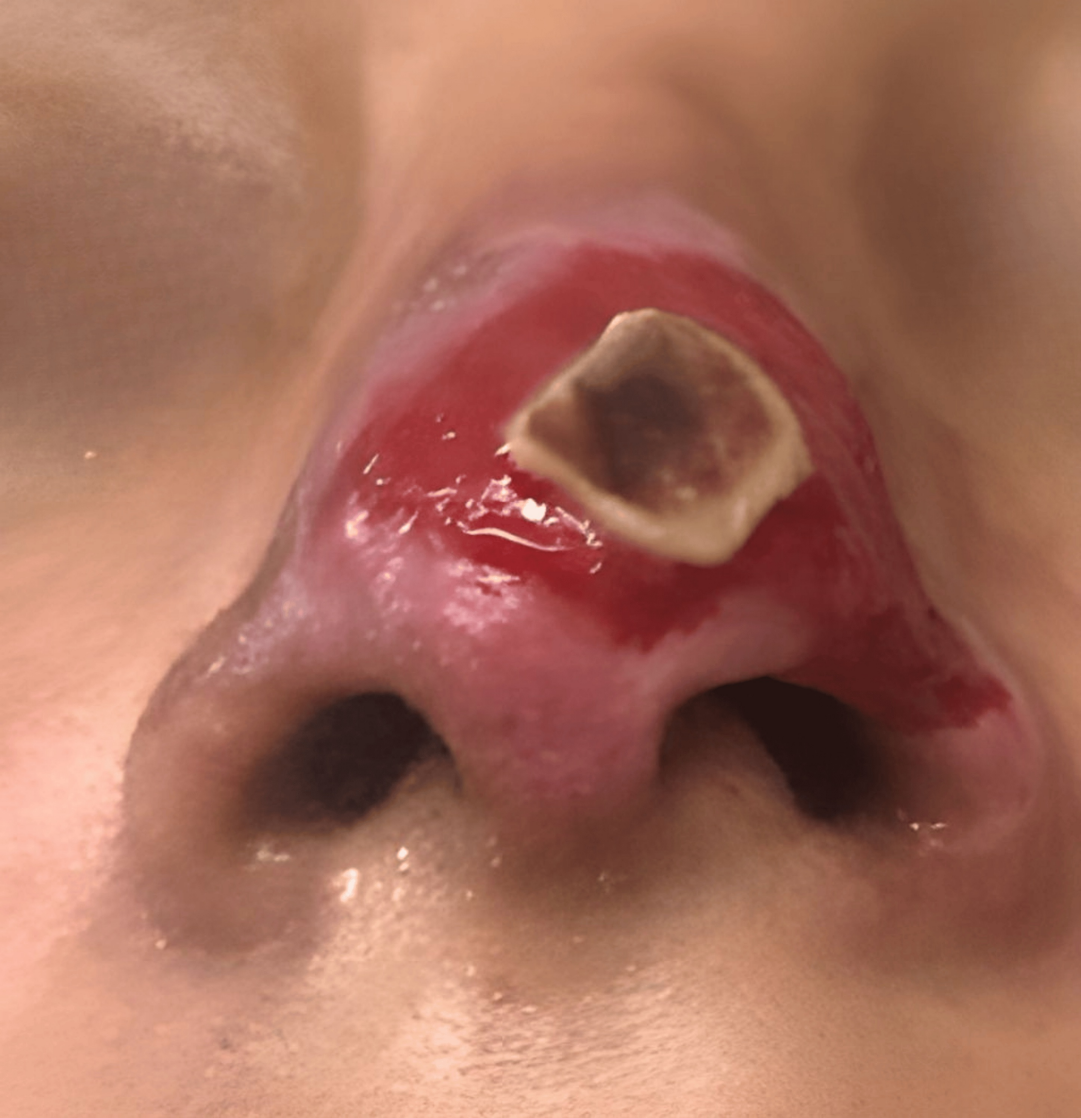
Clinical evolution on day 23 of follow-up (28 days after the initial procedure). Advanced eschar detachment with a broad reddish, moist granulation bed. A central necrotic fragment persists in spontaneous resolution, with incipient re-epithelialization at the edges and reduced peripheral erythema.

At the follow-up on day 23 (28 days after the initial injection), further detachment of the eschar was observed, with broader exposure of a reddish, moist granulation bed. A central fragment of necrotic tissue persisted, well delimited and in the process of spontaneous elimination, while the wound edges showed incipient reepithelialization and decreased peripheral erythema. The patient reported a notable reduction in pain and improvement in local sensitivity (Figure 5).

**Figure 6:**
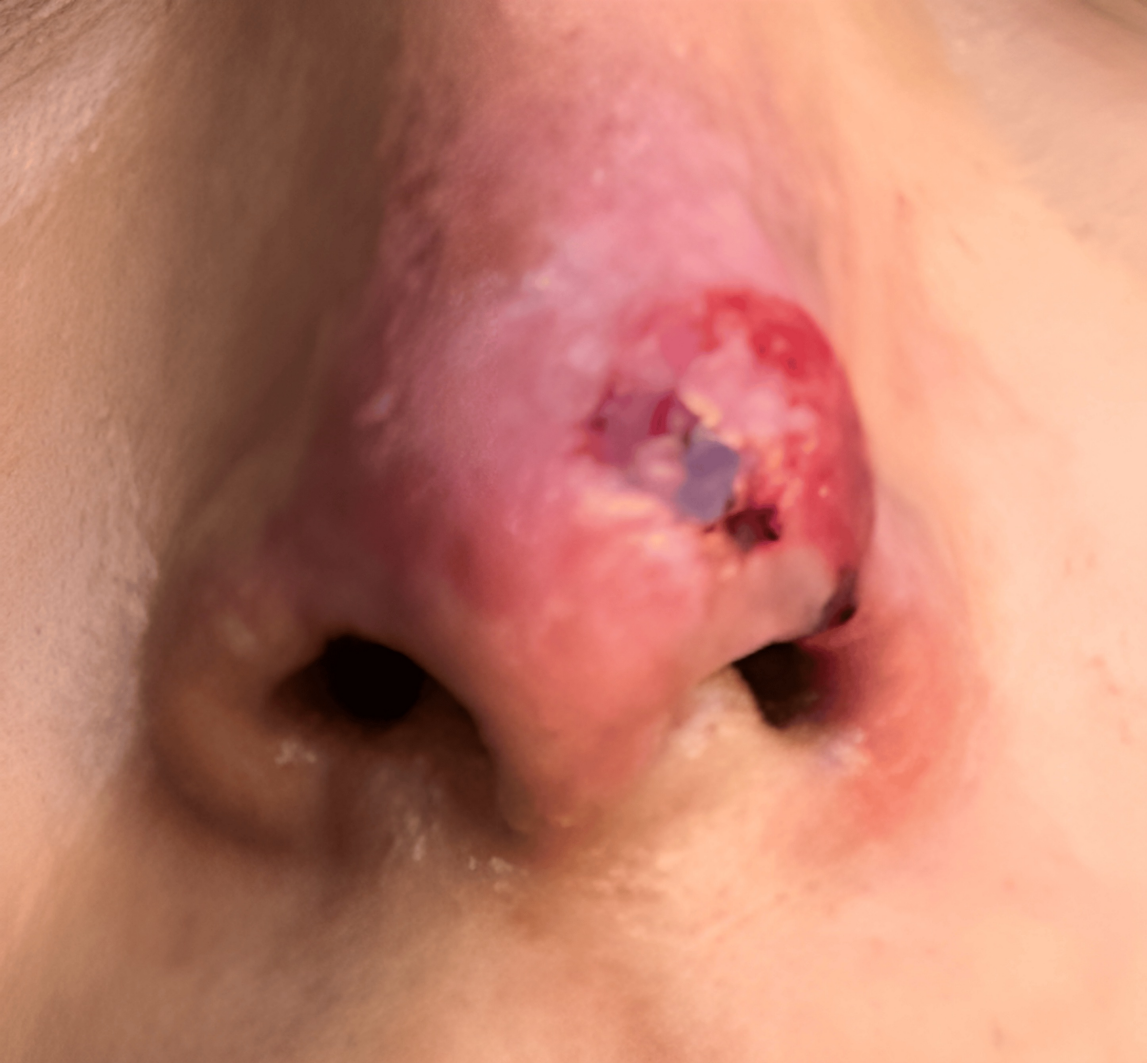
Clinical evolution at approximately the fifth week of follow-up. Predominant reepithelialization of the nasal tip is observed, with a small central area of immature tissue and residual crust, accompanied by mild peripheral hyperpigmentation, with no evidence of active infection.

At the follow-up conducted around the fifth week, the patient showed near-complete resolution of necrotic tissue, with predominant re-epithelialization of the nasal tip. A central area of immature tissue with minimal residual crust and slight peripheral hyperpigmentation persisted. Erythema was markedly reduced, and the patient reported the absence of pain (Figure 6).

**Figure 7:**
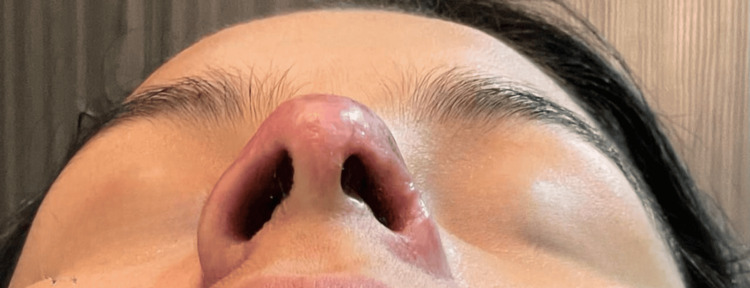
Clinical evolution on day 58 of follow-up. More advanced healing is observed, with stable epithelial tissue; however, a marked central depression persists, reinforcing the suspicion of cartilaginous damage.

**Figure 8:**
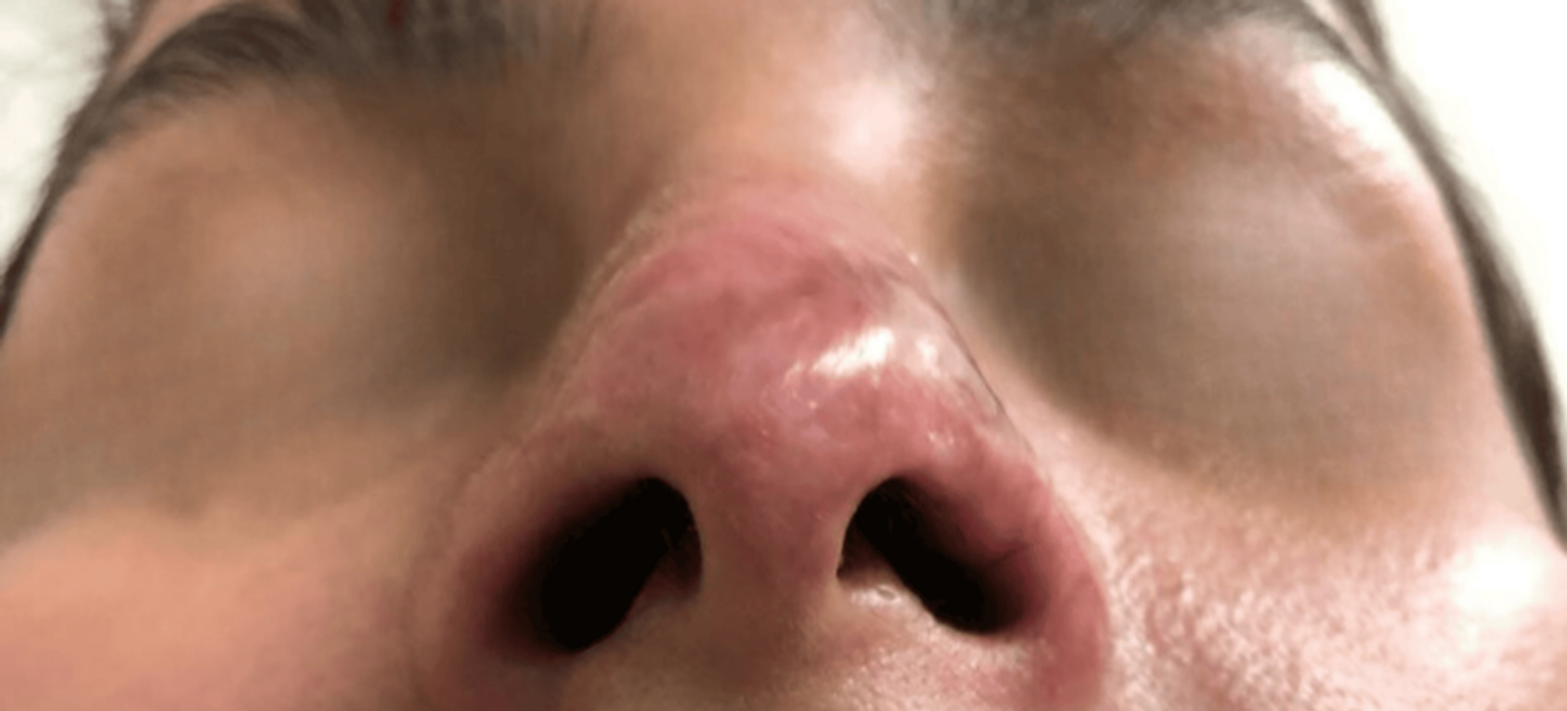
Clinical evolution on day 38 of follow-up (43 days after the initial procedure). The lesion shows greater epithelial coverage with reduced erythema, although a central depression and nasal asymmetry persist, consistent with cartilaginous involvement.

**Figure 9:**
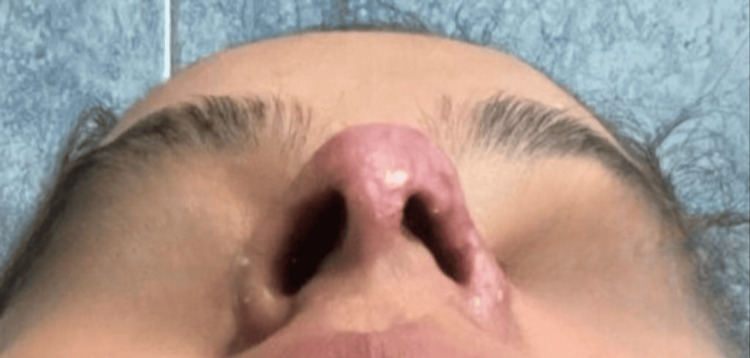
Clinical evolution on day 32 of follow-up. Progressive re-epithelialization of the nasal tip with persistence of a central depression and asymmetry, suggestive of cartilaginous involvement.

Between days 32 and 58 of follow-up, the patient demonstrated progressive re-epithelialization of the nasal tip; however, a persistent central depression and asymmetry were noted, suggestive of underlying cartilaginous involvement (Figures 7-9). Based on these findings, it was considered that although the secretome favored dermal regeneration and cutaneous healing, it did not exert a sufficient effect on the deeper cartilaginous tissue. Consequently, an application of MSCs (10 million cells) was administered with the aim of stimulating deeper regenerative processes.

**Figure 10:**
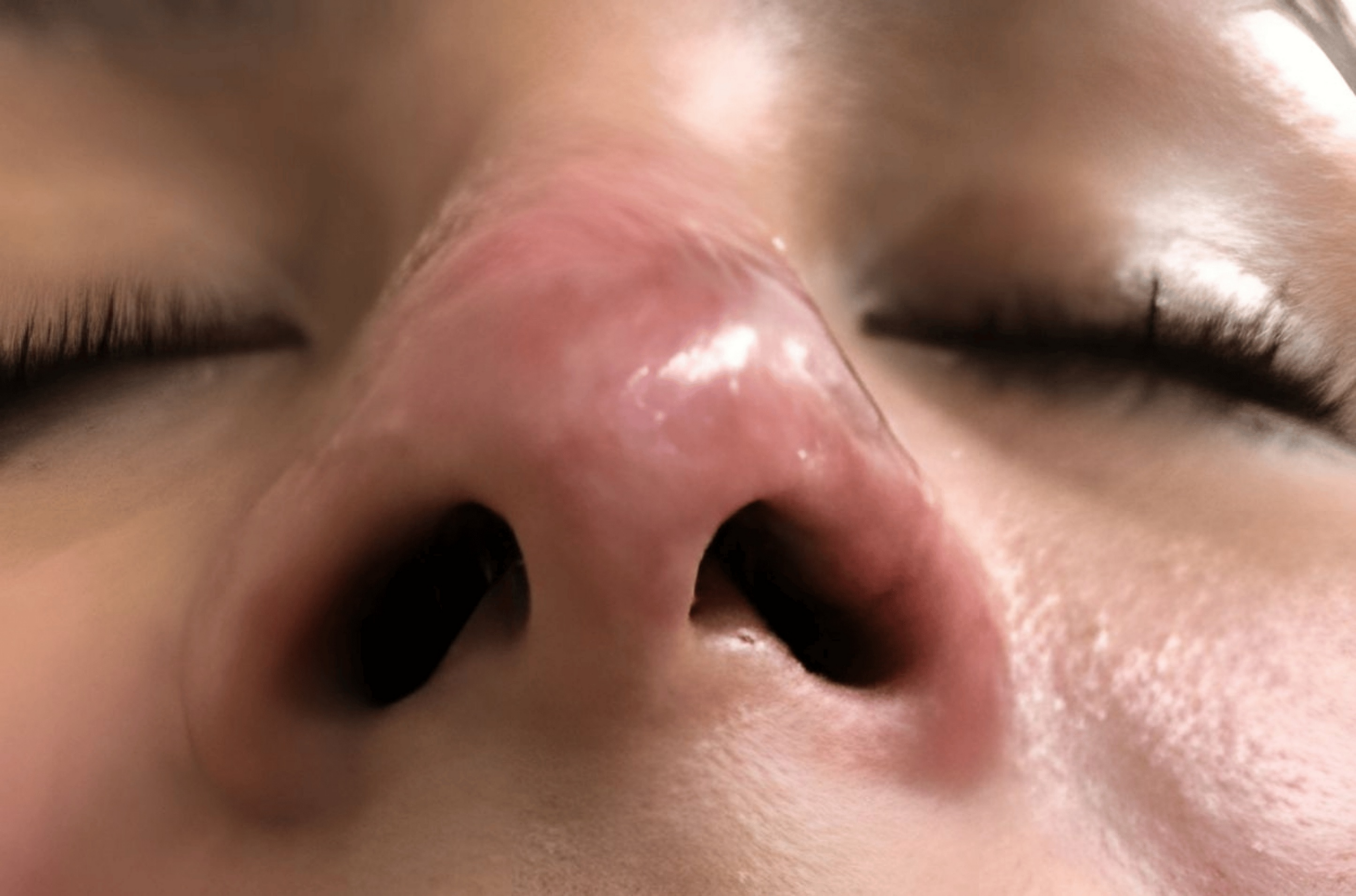
Clinical evolution 45 days after mesenchymal stem cell application. Complete closure of the defect is evident, with adequate cutaneous coverage and improvement of the nasal contour, although residual aesthetic sequelae remain

Forty-five days after this intervention, a cicatrized area with stable epithelial coverage was observed, along with evident improvement in nasal contour, although mild residual aesthetic sequelae persisted (Figure 10).

**Figure 11:**
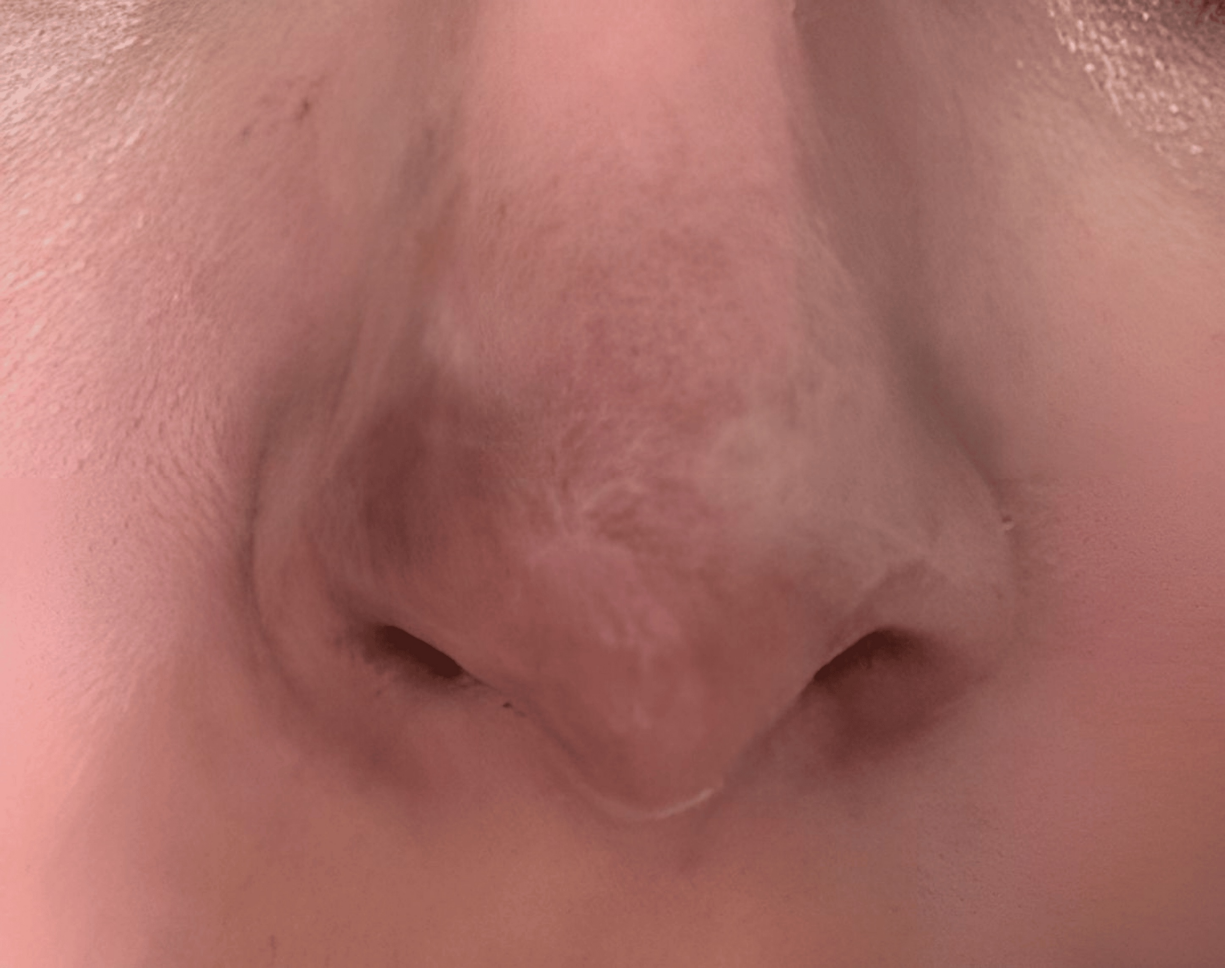
Clinical evolution at two-year follow-up The scar appears stable, with focal hypopigmentation and slight irregularity of the nasal tip surface.

**Figure 12:**
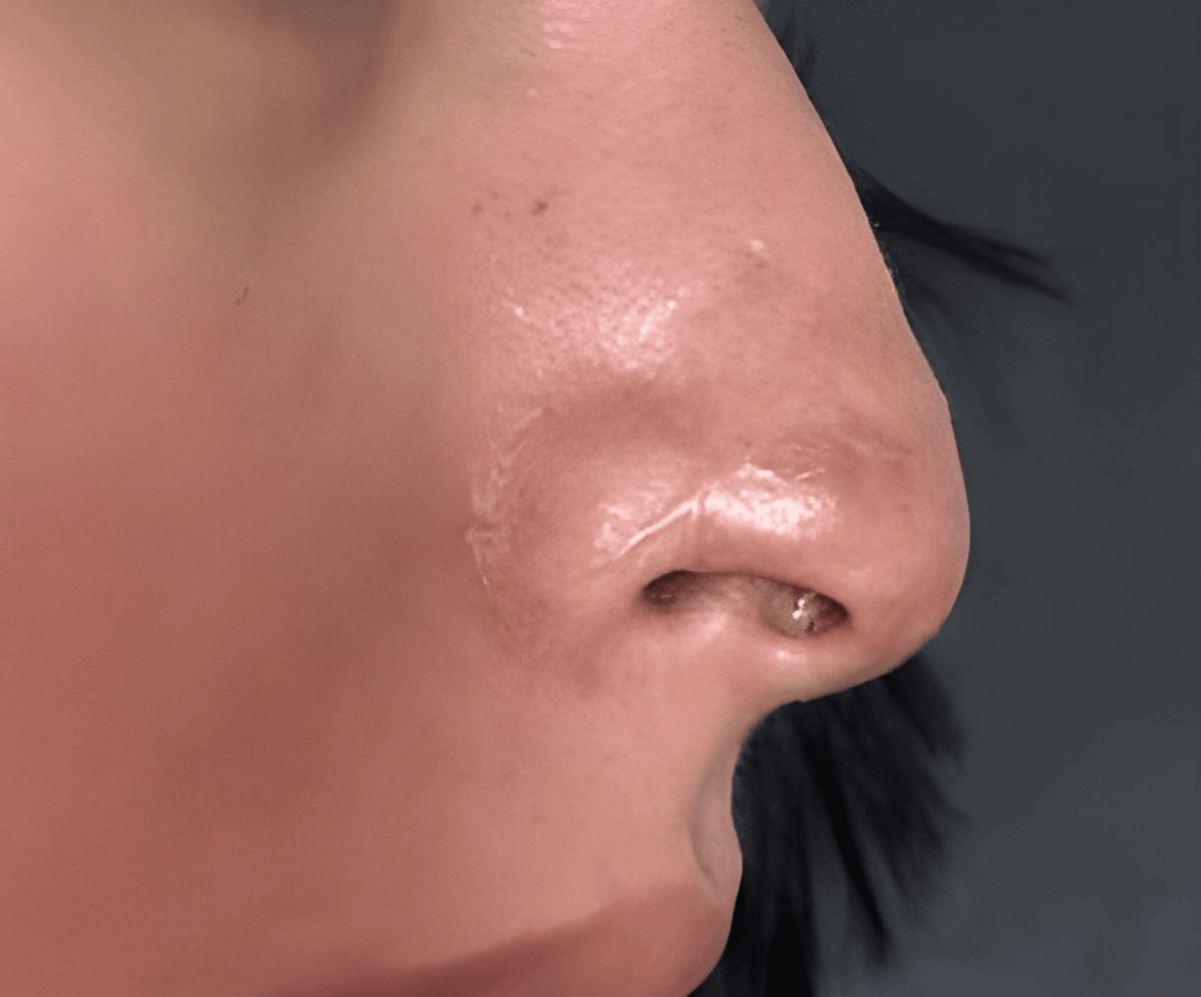
Lateral view at two-year follow-up. Cutaneous integration of the scar is observed, with mild irregularity in the nasal profile, without signs of recurrence or secondary complications.

At long-term follow-up, approximately two years after the initial event, the patient returned for evaluation. At that time, a stable scar was documented, characterized by focal hypopigmentation and mild surface irregularity at the nasal tip (Figures 11, 12). No signs of recurrence, secondary complications, or functional impairment were observed. At present, additional regenerative medicine strategies are being considered to address residual pigmentation changes and scar remodeling.

Throughout the healing process, the patient complemented medical management with topical home application of a non-pharmacological hydrating facial serum containing bioactive factors. No wound dressings, topical antibiotics, or advanced wound care products were used during the follow-up period.

At the end of follow-up, the patient achieved complete healing of the affected area, with preservation of nasal function and an aesthetic sequela characterized by focal hypopigmentation and mild surface irregularity. No functional impairment or late complications were identified. The clinical evolution and therapeutic interventions are summarized in Table 1, which chronologically outlines the key milestones of the management strategy and the outcomes obtained.

**Table 1:** Timeline of clinical evolution, therapeutic interventions, and outcomes.

Time from the initial procedure	Clinical event/Intervention	Clinical findings/Outcome
Day 0	Hyaluronic acid nasal filler injection by a non-medical injector	Onset of pain and discoloration at the nasal tip
Days 2–3	First clinical evaluation. Diagnosis of vascular occlusion	Erythema, pain, and early necrotic changes at the nasal tip
Day 1 of follow-up (first 24 hours)	Three hyaluronidase infiltrations (1,500 IU each, every 8 hours; total 4,500 IU), reconstituted in 0.9% saline solution. Oral sildenafil 50 mg daily for 3 days. Single low dose of prednisone 5 mg	Persistent pain, no progression of necrosis
Days 1–3 of follow-up	Intravenous peroxidation therapy: hydrogen peroxide 3.5% (250 mL saline) + 3 cm³ DMSO, twice daily for 3 days	Improved pain and perceived tissue oxygenation
Week 1 of follow-up	Secretome infiltrations: 3 doses during the first week	Improvement in skin color and onset of granulation tissue
Day 11 (day 6 at our clinic)	Clinical evaluation	Well-demarcated necrosis at the nasal tip, dry eschar, erythematous borders, localized tenderness, no signs of active infection (Figure 2)
Day 18 (day 13 at our clinic)	Clinical evaluation	Persistent necrotic eschar with early marginal detachment and incipient granulation tissue (Figure 3)
Day 25 (day 20 at our clinic)	Clinical evaluation	Partial detachment of the eschar; erythematous, moist wound bed with abundant granulation tissue (Figure 4)
Day 28 (day 23 at our clinic)	Clinical evaluation	Broad exposure of a reddish granulation bed; central necrotic fragment; early re-epithelialization (Figure 5)
Day 36 (day 31 at our clinic)	Clinical evaluation	Near-complete detachment of the eschar; homogeneous granulation bed; peripheral re-epithelialization (Figure 6)
Week 5	Clinical evaluation	Predominant re-epithelialization; minimal residual crusting; mild peripheral hyperpigmentation (Figure 6)
Days 32–58	Additional secretome infiltrations (total of 12 applications completed)	Progressive epithelialization; persistent central depression and asymmetry suggestive of cartilaginous involvement (Figures 7–9)
Day 58+	Mesenchymal stem cell application (10 million cells)	At 45 days: complete defect closure, stable cutaneous coverage, and improved nasal contour (Figure 10)
2 years	Long-term follow-up	Stable scar with focal hypopigmentation and mild surface irregularity (Figures 11–12)

## Discussion

Vascular complications associated with dermal filler use represent one of the most challenging scenarios in aesthetic medicine, with reports including cutaneous necrosis, structural loss, and permanent scarring [24,25]. Traditionally, management has been based on the immediate and repeated administration of hyaluronidase, in combination with adjuvant drugs, massage, and, in some cases, hyperbaric oxygen therapy [14,16]. However, even with early intervention, the risk of extensive necrosis, nasal deformity, and even septal cartilage loss remains, which has led reconstructive surgery to be considered the standard rescue option in advanced cases [1].

In the present case, a combined approach was used, integrating guideline-aligned measures (high-dose hyaluronidase and adjunctive therapy) with additional interventions intended to support tissue recovery. The favorable clinical course observed after the regenerative phase suggests that tissue restoration in selected cases may be achievable without immediate reconstructive surgery. Importantly, this report should be interpreted as hypothesis-generating rather than confirmatory evidence, as causality cannot be established in a single case, and multiple therapies were administered sequentially.

The combination of MSC-derived secretome and subsequent administration of viable stem cells offered a non-surgical strategy associated with functional preservation and cutaneous healing. The secretome, defined as the set of soluble proteins, growth factors, and extracellular vesicles released by stem cells, has demonstrated pro-angiogenic, immunomodulatory, and antifibrotic effects [10]. These actions may plausibly contribute to improved microcirculation and wound repair through pathways that include angiogenic signaling (e.g., vascular endothelial growth factor-related activity), modulation of inflammatory cascades, and remodeling of extracellular matrix, mechanisms described in the broader regenerative literature [10]. Recent studies have documented its ability to accelerate healing in chronic wounds and to modulate dermal regeneration by stimulating fibroblasts and reorganizing collagen [11,13].

In this context, secretome application in the early phases of the ischemic process may have favored granulation tissue formation and early re-epithelialization, reducing the extent of necrosis and supporting progressive wound closure. Literature supports this effect: in models of cutaneous ulcers and burns, secretome has promoted angiogenesis and accelerated repair without the need for grafting [12,26]. However, no direct clinical reports currently exist regarding its use in HA filler-related vascular complications; therefore, any extrapolation from wound and burn data must be interpreted cautiously, and the present observation should be viewed as preliminary.

Despite cutaneous improvement, persistence of a central depression and nasal asymmetry suggested deeper structural involvement, potentially including cartilaginous compromise. This highlights a limitation of secretome, since its effects are primarily described in soft tissues. Consequently, the decision was made to proceed with MSC administration, whose efficacy in cartilage regeneration has been investigated in contexts such as osteoarthritis and chondral defects [27]. Several studies have demonstrated that stem cells possess chondrogenic potential and stimulate extracellular matrix synthesis, thereby improving structural integrity [28-31]. In our case, cell application was temporally associated with improvement in nasal contour, supporting a possible reparative contribution at the structural level; nevertheless, cartilage restoration was inferred clinically, as no imaging or histopathological confirmation was available.

Notably, alternative autologous options such as stromal vascular fraction (SVF) have been proposed as potentially relevant in regenerative contexts due to their heterogeneous cellular composition and angiogenic paracrine activity. SVF was not used in this case because it was not available within our clinical setting at the time of management, and the therapeutic decision prioritized the standardized secretome protocol already accessible for serial local applications. Future reports and comparative studies could explore whether SVF or combined approaches offer additional benefit in complex ischemic injuries.

This report contributes to the literature by illustrating that a combined regenerative strategy may be feasible as an adjunctive, non-surgical approach in selected cases of nasal necrosis secondary to vascular occlusion, particularly when surgery is not desired or not immediately available. Nonetheless, it remains essential to emphasize that evidence is limited and that standardized validation requires controlled clinical studies with long-term follow-up and objective endpoints (e.g., healing time, scar quality, and structural assessment using ultrasound or MRI), in addition to transparent reporting of product sourcing and protocol details. Finally, because some adjunctive interventions used in this case are not standard of care for filler-related vascular occlusion, safety oversight, informed consent, and careful patient selection are critical considerations when exploring such approaches.

## Conclusions

This case illustrates the potential role of regenerative medicine as an adjunctive approach in the management of vascular complications from dermal fillers. The combined use of MSC-derived secretome and stem cells was associated with preservation of tissue viability and nasal function, and with progressive cutaneous and structural recovery in this patient. However, these findings should be interpreted as hypothesis-generating, rather than evidence of a proven alternative to reconstructive surgery. Further controlled clinical studies are required to define the safety, reproducibility, and clinical value of regenerative strategies in filler-induced vascular complications.
